# Pragmatism and integrated knowledge translation: exploring the compatabilities and tensions

**DOI:** 10.1002/nop2.30

**Published:** 2015-10-08

**Authors:** Lorelli Nowell

**Affiliations:** ^1^ Faculty of NursingUniversity of CalgaryCalgaryAlbertaT2N 1N4Canada

**Keywords:** Epistemology, evidence, knowledge translation, nursing knowledge, nursing research, philosophy, pragmatism

## Abstract

**Aim:**

This paper presents a discussion of the role of the philosophy of pragmatism in the integrated knowledge translation approach to research.

**Design:**

Critical inquiry is used to discuss bringing pragmatic philosophy and the integrated knowledge translation approach to research together to advance nursing knowledge.

**Methods:**

This paper draws from the literature written on the philosophy of pragmatism and from the current literature on knowledge translation. The possibilities, tensions and limitations for underpinning an integrated knowledge translation research approach with pragmatic philosophy are discussed while highlighting the implications this has for creating knowledge aimed at advancing the practice of nursing.

**Results:**

The implications for how nursing knowledge is created in using an integrated knowledge translation approach that is underpinned by pragmatic philosophy are important. Creating nursing knowledge that address the complex problems found in nursing practice is needed. In acknowledging the inseparability of knowledge and practice, researchers, practitioners, policy makers and the public can come together to co‐create knowledge that is useful for the practice of nursing. It is these implications of underpinning an integrated knowledge translation research approach with pragmatic philosophy that are significant in creating nursing knowledge that advances the practice of nursing.

## Introduction

Nursing knowledge development is largely understood as generating evidence to advance the practice of nursing (Risjord 2010). Evidence‐based nursing knowledge is needed in today's complex environments and research designs that can meet these complex problems are required. Acknowledging the inseparability of knowledge and practice provides opportunities for researchers, practitioners, policy makers and public to co‐create knowledge that is useful for the practice of nursing. Using a research paradigm which is able to embrace these complexities and offer new insights to influence nursing practice is of considerable importance for nursing (Carr 2009).

Benner *et al*. (2010) call for nursing research designs that reflect the multidimensionality and complexity of practical nursing knowledge. An Integrated knowledge translation (iKT) approach to research is one way to answer this call as knowledge created from multiple paradigms is integrated to increase the cumulative relevant research based knowledge provided to nurses (Weaver & Olson 2006). Integrated knowledge translation (iKT) is an approach to research that aims to address a need identified through collaboration between researchers, practitioners, policy makers and the public to create and implement knowledge to improve efficiencies in the health care system (Graham *et al*. 2006). As researchers choose to use iKT approaches, they will continue to embrace comprehensive understandings of evidence and the complex context where nurses practice, where researchers, practitioners, policy makers and the public can work together to create nursing knowledge and translate this knowledge into practice (Kirkham *et al*. 2007). The philosophical underpinning of pragmatism may allow nursing researchers the opportunity to use dynamic approaches to address the complex and multifaceted research problems often encountered in nursing practice (Doyle *et al*. 2009).

Knowledge translation (KT) has been defined by the Canadian Institute of Health Research (CIHR) (2014) ‘as a dynamic and iterative process that includes synthesis, dissemination, exchange and ethically sound application of knowledge to improve the health of Canadians, provide more effective health services and products and strengthen the health care system’. Despite its significance, little has been written about the philosophical underpinnings of the iKT approach to research, opening up important areas for further philosophical consideration. The purpose of this article was to help address this gap in literature by discussing the compatibilities and tensions in bringing pragmatic philosophy and the iKT approach to research together to advance nursing knowledge.

## Integrated knowledge translation as an approach to nursing research

The CIHR KT framework, developed by Graham *et al*. (2006), offers a global picture of the overall KT process as integrated in the research knowledge production and application cycle. This KT framework provides an approach that brings together planned action theories, where knowledge is exchanged between relevant stakeholders and results in action. KT is generally understood as consisting of two concepts, knowledge creation and knowledge implementation. In reality, the process of KT is complex and dynamic, where the boundaries between knowledge creation and implementation are fluid and permeable (Straus *et al*. 2009a). In the CIHR KT framework, knowledge creation consists of considering all types of knowledge where researchers tailor their research questions and activities to address problems identified in collaboration with practitioners, policy makers and the public to best meet their needs. Knowledge implementation represents activities in applying knowledge in practice, including the following: identifying the problem; identifying, reviewing and critically appraising relevant knowledge; adapting the knowledge to the local context; assessing the context relevant barriers and facilitators of implementing the intervention; selecting, tailoring, implementing and monitoring the intervention; evaluating outcomes or impacts of the intervention; and determining strategies for ensuring sustained use of the evidence‐based intervention.

Although the CIHR KT model offers an overall view of KT process, the use of other models and/or frameworks that represent an individual user's perspective, as well as those that address contextual factors may be necessary to successfully implement specific processes in the CIHR KT model.

Effective iKT requires not only the environmental organizational view, but also the micro‐perspective of individual stakeholders (Davis 2005). Other models and frameworks may be used to augment understanding of the specific KT components and contextual factors to be considered to facilitate successful iKT processes including the development of effective communications and partnerships with stakeholders.

The Understanding‐User‐Context Framework (Jacobson *et al*. 2003) for knowledge translation provides practical guidelines that can be used by researchers to establish interactions and engage stakeholders in the knowledge translation process. Context focused models and frameworks, such as the Ottawa Model of Research Use (Logan & Graham 1998), the Promoting Action on Research Implementation in Health Services Framework (Rycroft‐Malone 2004) and the Coordinated Implementation Model (Lomas 1993) can be used to understand the contextual factors that may contribute to the success or failure of KT efforts. Individual‐focused models such as the Stetler Model of Research Utilization (Stetler 2001) is a practitioner‐oriented model that offers a procedural and conceptual guide for the application of research in practice while considering the practical aspects of clinical decisions. These models and frameworks offer a deeper understanding of contextual factors to be considered to facilitate successful iKT processes.

Despite the rapid uptake of iKT, approaches to iKT are still being developed and the philosophical grounding of this approach remains a relatively neglected area of discussion in the literature. One concept that the iKT research movement has been linked to is the ‘paradigm shift,’ as popularized by Thomas Kuhn (Hedges 2007, Reimer‐Kirkham *et al*. 2009). The iKT paradigm shift has been described as a move from viewing knowledge as constituting only what is observable and context‐stripped to integrating context‐sensitive knowledge and recognizing diverse ways of generating knowledge that are considered legitimate evidence (Reimer‐Kirkham *et al*. 2009). The paradigm shift is furthered through viewing knowledge as a process and knowledge creation as complex, as opposed to knowledge as a product and knowledge creation as a linear rational process (Reimer‐Kirkham *et al*. 2009). The role of the researcher has also shifted from being a producer and interpreter of knowledge to being an involved collaborator, who develops community, clinical and policy‐maker partnerships to identify, co‐construct and consider multiple sources of evidence (Poole 2008, Reimer‐Kirkham *et al*. 2009).

## Basic principles of pragmatic philosophy

Researchers who use an iKT approach to research may also draw on pragmatic philosophy to create nursing knowledge that advances the practice of nursing. For pragmatists, human values, visions, actions and interactions precede the search for descriptions and explanations. Pragmatic research is motivated by anticipated outcomes and the choice of what to research and how is broadly conditioned by where we want to go (Cherryholmes 1992). The common ground for three pragmatic philosophers of note – Peirce, James and Dewey – is the emphasis of practical usefulness and consequences of ideas and statements.

The founding father of pragmatism, Charles Sanders Peirce, presented truth as an understanding of reality from an empirical point of view. He described pragmatism as a method of using scientific logic to clarify the meaning of concepts or ideas through investigating their potential relationship with the real world. The word pragmatism was formed to express a maxim of logic intended to deliver a method for the analysis of concepts, where the ‘conceivable practical consequences’ were traced out (Peirce 1905, 494). Peirce (1878), argued that conditional statements generated for a concept or idea should list the practical outcomes we can expect from them. William James moved pragmatism away from Peirce's scientifically founded philosophy to an approach that explored the consequences of beliefs. James (1907) argued that there can be different kinds of truth and those ideas and beliefs become true just in so far as they help people to get into satisfactory relations with other parts of their experience. James (1907 p.26) believed that: ‘all realities influence our practice and that influence is their meaning for us.’ For James, truth was determined by asking how the world be different if an alternative was found to be true. If nothing would be different that the alternative did not make sense (1907). Dewey later brought a more radical perception of pragmatic inquiry, where he promoted practical problem solving. Dewey (1931) argued that pragmatism did not exist on antecedent phenomenon but on consequent phenomenon and the possibilities of action. Dewey's change in point of view was revolutionary in that general ideas should not simply report past experiences but should instead be the bases for organizing future observations and experiences (1931).

## Integrated knowledge translation and pragmatism

### Knowledge and knowledge creation

In adopting a pragmatic philosophy, knowledge is understood as being constructed based on the reality of the world we experience and live in and encompasses not only the reality of the past but also what is possible to create for the future. The knowledge one has and the quality of believing this knowledge to be true depends on one's real world experience and interests. Knowing in a complex reality, such as clinical nursing practice settings, requires multiple perspectives to be considered, where knowledge is not necessarily always convergent but might be varied, or even contradictory. In recognizing diverse ways of knowing as legitimate truths, the depth and breadth of these multiple truths can lead us to a greater understanding of larger complex truths. Bringing together various sources of knowledge, with the aim of creating a deeper understanding of phenomenon of interest, is a way to study complex problems that may exceed an individual's capabilities of understanding a phenomenon independently.

A pragmatic viewpoint offers epistemological justification for bringing together multiple sources of knowledge with the goal of finding workable solutions, gaining a greater understanding of people and the world in which we live and practice and solving individual and social problems (Johnson & Onwuegbuzie 2004). By exploring the differences in knowledge that researchers, practitioners, policy makers and the public bring forth on a problem, the iKT approach to research supports creating knowledge that is more insightful than knowledge that is created individually or that which is produced by research only (Rycroft‐Malone 2008). Subsequent knowledge created through this process is then put into practice through cooperative action between the researchers, practitioners, policy makers and the public (Rycroft‐Malone 2008, Reimer‐Kirkham *et al*. 2009).

### Truth is what works

The practice of research consists of a continual search to create new knowledge or truths to improve practice. The mantra of pragmatism is ‘truth is what works,’ where truth is always considered fallible, provisional and revisable as it is only considered truth while it works best (McCready 2010, p. 192). As with the iKT approach to research, current truths and knowledge are tentative and change over time. Knowledge that is obtained through an iKT research approach is rarely if ever viewed as certain or absolute. Knowledge is instead selected, tailored and adapted to meet the needs of the local context to determine what works best.

From a pragmatic perspective, the truth of an idea is not stagnant, rather truth happens to an idea in the course of experience (James 1907). Truths infinitely emerge from facts that are added to again and again to create or reveal new truth (James 1907). The process of verifying truths involves setting ideas and theories to work in everyday practice experiences and determining their value to a particular situation in terms of consequences (Doane & Varcoe 2005). Similarly, the action cycle of iKT research approach is the process of putting knowledge into action in everyday practice. How knowledge is used in practice is assessed and the impacts and outcomes of using the knowledge are evaluated to determine its value. If the knowledge is of little value, further adjustments can be made to refine the knowledge to make it more useful. Similarly, from a pragmatic perspective when something is not working further discussion and investigation can identify errors and attempts can be made to address these barriers (Hannes & Lockwood 2011).

In pragmatic philosophy and in the iKT approach to research, the process of knowledge creation is an infinite loop. Knowledge becomes a process rather than a product and knowledge creation becomes a complex rather than linear process (Reimer‐Kirkham *et al*. 2009). In an iKT research approach, researchers, practitioners, policy makers and public continually try to improve on past understandings in a way that works in the context where they practice (Onwuegbuzie *et al*. 2009). Knowledge is constantly being adapted in where researcher's works with practitioners, policy makers and the public to continually try to improve practice by understandings what works in practice. A pragmatic perspective acknowledges the inseparability and interdependence of knowledge and practice and recognizes the integral role practice experiences play in ongoing knowledge creation (Doane & Varcoe 2005). I argue that the iterative process of knowledge creation is required to generate and adapt new knowledge that is useful in answering ever‐changing and complex practice problems.

### Communities of practice

Knowledge‐to‐action gaps exist when the knowledge created does not address diverse practice demands, which may be a knowledge creation problem (Van De Ven 2013) that demands for bridges to be built between researcher and practitioners forming relevant communities of inquiry (Hannes & Lockwood 2011). Dewey (1931) identified communities of inquiry, including all those interested in resolving a problem, as one of the major building blocks of pragmatism. The collaborative process between researchers, practitioners policy makers and public is a hallmark of iKT, whereby the researcher is an involved collaborator, negotiator and communicator who develops partnerships that identify, co‐construct and consider multiple sources of evidence (Poole 2008, Reimer‐Kirkham *et al*. 2009). Promoting participation and maintaining relationships between researchers and practitioners is integral to developing collaborative knowledge that will be effective in practice (Poole 2008, Grimshaw *et al*. 2012).

Mutual understanding between researchers and practitioners as they relate to the processes of communication and shared meaning are central to the pragmatic approach (Morgan 2007). Poole (2008) suggests that these ‘communities of practice’ uniquely combine researchers who create knowledge, a community of people who care about the knowledge and the shared practice for which they are developing knowledge. I argue that communities of practice are needed to translate knowledge across the research and practice boundaries and that forming relationships between researchers, practitioners, policy makers and the public is needed to have an impact on advancing the science and practice of nursing.

## Philosophical tensions with an iKT research approach

Despite the identified compatibilities of underpinning the iKT approach to research with a pragmatic philosophy of science, there are tensions in doing so that could benefit from further exploration. The way knowledge is viewed and what is determined to be legitimate knowledge may be regarded as a possible tension between iKT and pragmatic philosophy. How knowledge is viewed carries significant implications for how an iKT research approach is envisioned and ultimately evaluated. Much of the academic literature has described the knowledge‐to‐action gap as an epistemological problem and emphasized the importance of integrating sound knowledge into practice. Doane and Varcoe (2008), argued that the knowledge‐to‐action gap may be promoted by the kinds of knowledge that is considered valid evidence and that which practice is subsequently based. The privileging of empirical knowledge may limit consideration of evidence from a variety of other research methodologies and failing to address the complexities and realities of practice (Doane & Varcoe 2008). Although I acknowledge this as a potential tension, I believe that as the iKT approach to research continues to embrace broader interpretations of evidence, knowledge development and translation will continue to work towards addressing complex practice problems.

When using an iKT approach to research, the process of knowledge creation is an infinite loop where knowledge is viewed as a complex process rather than a product. Knowledge is selected, tailored and adapted through complex and iterative cycles to illicit a change in practice to meet the needs of the local context. Reimer‐Kirkham *et al*. (2009), highlighted this as a potential tension and thus suggested that the emphasis on evaluating the success of iKT on the basis of practice changes could be understood as viewing knowledge as a product rather than a process. This may be reflective of the ongoing tension between research and practice. I counter this argument in suggesting that considering practitioners, policy makers and the public throughout the research process is integral to an iKT approach. Promoting participation and maintaining relationships between researchers, practitioners, policy makers and the public develops collaborative knowledge synthesis, analysis and exchange where knowledge is co‐created through a complex process.

Tension can be assumed when the relationship between knowledge and practice is viewed from an ontological perspective – a perspective not adopted by many pragmatic philosophers. Doane and Varcoe (2008) viewed ontology as central to how we translate knowledge into action. They argued that understanding, including interpretation and translation, is not simply a mode of knowing but a way of being and a way of relating (Doane & Varcoe 2008). From this perspective, the central focus of iKT should not be how to get knowledge used in practice because it is understood that practice is already evidence based. Rather, Doane and Varcoe (2008) argued that using an iKT approach should focus on inquiring into one's way‐of‐being and how this way‐of‐being shapes the connection between knowledge and practice. An ontological understanding of the connections between knowledge and practice highlights that iKT approaches should be guided by how knowledge created can inform the possibilities for being with and responding to particular practice situations. Understanding and enacting the interconnection of theory, evidence and practice requires examining how epistemology and ontology are intricately intertwined in practice (Doane & Varcoe 2008). I do not deny that metaphysical problems exist in the iKT research approach; however, in taking a pragmatic philosophical stance rather than engage in meta‐theoretical debating, I chose to pursue a pragmatic approach to research in hopes of seeking knowledge to answer relevant practice problems (Friedrichs & Kratochwil 2009).

## Limitations of pragmatic philosophy

Many current philosophers have rejected pragmatism as a philosophy because it chooses not to engage in meta‐theoretical debating as a solution to many philosophical debates (Johnson & Onwuegbuzie 2004), instead choosing to pursue knowledge that will enable us to deal with relevant problems (Friedrichs & Kratochwil 2009). Pragmatism has also been criticized for focusing on practical results and ignoring philosophy and theory (McCready 2010). As a practitioner, not a philosopher of science, I understand the yearning to conduct research rather than argue about the philosophy that underpins it. To become a reflective and responsible scientific inquirer, I also acknowledge the need to understand the implications of the philosophical underpinning of my scientific practice. Pragmatists have a high regard for reality and the influence of this reality has on experiences. Morgan (2007), suggested that pragmatists do not deny that ontological problems exist, instead the pragmatist approach rejects the favouring of ontological assumptions, believing they are too narrow to approach philosophy of knowledge.

The ongoing paradigm debate over the place and value of quantitative and qualitative research methodologies continues today in social science research. Pragmatists have been criticized for considering the research question to be more important than either the method or the paradigm that underlies it (Doyle *et al*. 2009). Pragmatists reject the traditional dualist paradigms and instead endorse eclecticism and pluralism where methodological choice is based on the need to answer a research question rather than on philosophical alignment (Onwuegbuzie *et al*. 2009, Glogowska 2011). Pragmatic researchers argue that research paradigms can not only remain separate, but they also can be combined into another research paradigm (Onwuegbuzie *et al*. 2009). Although many might consider this a limitation of pragmatic philosophy, I believe that there is strength in combining existing research traditions in a pragmatic fashion to explore more complex practice problems.

The pragmatic process involves verifying what works through setting ideas and theories to work in everyday practice experiences (James 1907). A further criticism of pragmatism is that basing methodological choices solely what works does not answer the question for who is this working and to what end (Doyle *et al*. 2009). I believe that pragmatic researchers can address this limitation by explicitly acknowledging for who and how well the research is meant to be useful (Johnson & Onwuegbuzie 2004). Although many philosophers have rejected pragmatism due to the previously identified limitations, many have chosen pragmatic philosophy to underlie their scientific practice. In questioning the possibilities and limitations of this philosophy, a greater understanding of the implications for choosing pragmatism has been established. Consideration of the key tenets of pragmatic philosophy has illuminated how this philosophy is able to inform an iKT approach to research.

## Limitations of an iKT approach to research

Examining iKT though various philosophical lenses exposes certain limitations of this research approach. Ontological concerns have already been raised in regards to how iKT aims to translate knowledge into action. The assumption that knowledge can be packaged and translated is increasingly being cited as a limitation to iKT (Reimer‐Kirkham *et al*. 2009). Valid concerns have also been raised that by strictly or uncritically adhering to iKT tools, the rich contextual issues that influence practice may be stripped away. Problems may arise when iKT tools are seen as driving clinical decision making, rather than used as a tool to be used to assess and make decisions about best practices (Kirkham *et al*. 2007). While the use of iKT tools are a beginning point for guiding practice, I argue that their usefulness is dependent largely on the clinical judgment used to interpret evidence and make decisions about best evidence in context of, or as part of professional practice.

Given the complexity of practice, Kirkham *et al*. (2007) suggested the iKT perspective has considerable merit but different ways of looking at evidence are needed to broaden the scope of how knowledge is created. In iKT research, value is assigned to all knowledge including but not limited to qualitative, quantitative and experiential knowledge. As researchers who use an iKT approach continue to consider broader ways of viewing and interpreting evidence, knowledge creation and translation of this knowledge into action will attempt to answer complex practice problems.

Given that the purpose of KT was ultimately to improve the health care system through synthesis and ethically sound application of knowledge, many argue that thought should be given to what knowledge should be translated (Graham & Tetroe 2007). While the process of engaging in iKT is important, cautious translation of research into practice and policy must be ensured. Straus *et al*. (2009a) advised that working together with patients, public, clinicians and policy makers to ensure that the knowledge and its subsequent implementation are relevant to their needs. Despite these limitations, conducting research using an iKT approach has important implications for the development of knowledge to advance the practice of nursing.

## Implications for nursing

The tenets of pragmatism offer an attractive philosophical framework for an iKT research approach. Underpinning iKT with pragmatic philosophy has significant implications for how nursing knowledge is created. A pragmatic approach to iKT allows for the consideration of multiple theories, ideas and perspectives with a focus on the usefulness of this knowledge to create new knowledge (Doane & Varcoe 2005). The knowledge created through using an iKT approach can be used to help understand and support that which takes place in practice. The nursing knowledge created can be then effectively evaluated through the pragmatic approach by examining what works in practice (Carr 2009, McCready 2010).

Perhaps one of the most significant implications of a bringing together iKT and a pragmatic approach is the inclusion of practicing nurses, policy makers and the public in the knowledge creation process. A pragmatic perspective acknowledges the inseparability of knowledge and practice and recognizes the integral role practice experience plays in ongoing knowledge creation (Doane & Varcoe 2005). Researchers engaging in iKT approached recognize the capacity of all nurses to develop knowledge that addresses everyday practice challenges and to create and re‐create knowledge for practice. I argue that iKT promotes participation between researchers and practitioners and this participation is integral to developing collaborative knowledge that will be effective in practice.

The implications for how nursing knowledge is created in using an iKT approach that is underpinned by pragmatic philosophy are important. Creating nursing knowledge that address the complex problems found in nursing practice is needed. In acknowledging the inseparability of knowledge and practice, researchers, practitioners, policy makers and the public can come together to co‐create knowledge that is useful for the practice of nursing. It is these implications of underpinning an iKT research approach with pragmatic philosophy that are significant in creating nursing knowledge that advances the practice of nursing.

## Conclusion

Choosing the philosophy that will underlie ones research practice has implications and this choice is significant. In this paper, a philosophical discussion on the implications of choosing pragmatic philosophy to underpin the research practice of iKT was presented. The possibilities and tensions in bringing this philosophy and research practice together were explored highlighting the implications this has for creating nursing knowledge aimed at closing the knowledge‐to‐action gaps identified in practice and, therefore, advancing the practice of nursing. I have come to understand this is not a task for the faint of heart as choosing pragmatic philosophy to underpin iKT research means the present is always a new starting point and the creation, application and consideration of new knowledge is an iterative and never ending endeavour.

## Conflict of interest

No conflict of interest has been declared by the author.

## Author contributions

All authors have agreed on the final version and meet at least one of the following criteria [recommended by the ICMJE (http://www.icmje.org/recommendations/)]:

- substantial contributions to conception and design, acquisition of data, or analysis and interpretation of data;
- drafting the article or revising it critically for important intellectual content.
